# Indents

**Published:** 1914-04-15

**Authors:** 


### INDENTS.

☞Brief Articles of Technical or Scientific Value will receive notice and comment. Contributions invited.

*Why not Stamp out Typhoid?* We are pleased to see *The Journal Lancet* take a positive position on typhoid immunization as shown by the following statement from the December 1, 1913, editorial. "Typhoid can be stamped out with a hypodermic syringe filled with a non-irritating and safe vaccine. Hog cholera, a similar disease, can be controlled by the same means, but the serum must be as carefully prepared as typhoid vaccine. The administration should be as carefully done as on the human. Will the legislature see that the man is superior to the hog and make its appropriations with due care and respect?"

In the November, 1912, number of the BACTERIAL THERAPIST, we proposed editorially a method of typhoid eradication by immunization. Since then we have given this subject extensive, careful consideration and are still more convinced of its practical application. Typhoid germs will not remain alive long in water or the soil during warm weather, not over two or three months, and the only way typhoid germs are perpetuated is by replanting the soil or water with fresh germs from human excreta. These germs continue to contaminate the food supply with the resulting new crop of typhoid cases to reinoculate the soil and water. In ice, however, they have been known to remain alive for six months.

We do know that typhoid immunization with typhoid vaccine will protect against contracting the disease for at least one year, and in all probability for two or three years. From this it is perfectly clear that if a general typhoid immunization over the entire country were carried out during a winter season, not enough typhoid cases would develop during the following summer to replant the soil or water and consequently the germ would die out and become extinct.

Here the only other factor that remains to be considered is the typhoid carrier. The great difficulty at the present time is to find the typhoid carrier. By a general immunization the typhoid carriers would receive the immunizing doses and in that way a large percentage would become sufficiently immunized to cease being carriers, and if later on cases of typhoid fever should develop it would be a comparatively easy matter to trace the trouble to a carrier that had not become immune.

It is not more difficult to immunize the entire nation than to immunize a city, county or a state. Co-operation and concerted action is all that is necessary. Each community will take care of itself. If the government will make and furnish the vaccine, the expense will be nominal.

With the general public thoroughly informed as to what has been accomplished in the army, and that the purpose of the movement is to get rid of the disease entirely, there will be no objections, but on the contrary general immunization will be demanded.—*The Bacterial Therapist.*

*Statistics on Dental Caries.* Dr. R. Kloeser, of Marburg, Germany, gives in the first chapter of a most painstaking and comprehensive paper, an historical review of the publications on dental caries statistics that have heretofore appeared. The results of the examination of 519,318 German school children are given in sixteen tables, and the establishment of a central bureau of statistic is urged. The percentage of children suffering with dental caries is calculated to be 94.12 per cent., the average number of carious teeth being 7.19. Calculations as to the comparative distribution of caries in the two dentitions show that, of the above average number of carious teeth, four are of the deciduous, three of the permanent set, six belonging to the molar, one to the incisor class.

Examinations of 33,000 boys and as many girls proved that both sexes are alike in regard to susceptibility to caries, with the difference that girls show better deciduous, but worse permanent sets. The investigations of fourteen observers tend to show that the comparative frequency of caries in the upper and lower jaw is as 6.9 to 5.6, 552 of 1000 carious teeth being uppers, 448 lowers. Again the ratio of distribution of caries in the various kinds of teeth is 396 upper and 420 lower molars and premolars and 155 upper and 29 lower anterior teeth per 1000.

The lowest frequency of caries is found during those periods in which the majority of teeth are developed, viz: at the ages of three, six, and twelve. Beyond the age of fifteen, however, the present statistics are too unreliable to permit of indisputable conclusions.

The writer then refutes some of the statements made by Rose, whose figures do not tally at all, thus detracting greatly from the merits of his investigations. The relationship between dental caries and development of the human organism is established beyond doubt, and can be proved by figures, deterioration of the teeth involving loss of weight or lack of development. In a great many cases, nervous symtoms and eye and ear disease can be traced to dental troubles. In regard to the effects of caries upon mental efficiency, the author again attacks Rose, whose findings in this respect, together with those of American investigators, he stigmatizes as unscientific and not to be taken seriously. How very open to discussion this chapter is may be gathered from the findings of Wurfschmidt (see "Dental Diseases Among School Children and Their Effects upon Efficiency," DENTAL COSMOS, December 1913, p. 1292), whose methods seem no less scientific than those of Kloeser, and who has shown some very convincing statistics regarding this phase of the problem of caries. That the fitness for military service is directly influenced by dental conditions is not surprising, although in the writer's opinion it cannot be proved by numerical tables that, as Rose writes in his rather uncritical enthusiasm—"The better the teeth, the greater the body weight, the larger the chest, and the greater the fitness for military service." On the other hand, the general average of fitness for service is undoubtedly greatly raised by rational dental treatment during childhood and adolescence.

The most surprising feature of Kloeser's statistics is the finding that, despite the apparently universal spread of the oral hygiene movement throughout the empire, and the exemplary facilities for the dental treatment of German school children, of 51,778 children with carious teeth only 744, viz, 1.43 per cent., enjoy dental treatment, which means that only every seventieth child has done something toward the preservation of his teeth. Of 1,787,571 carious teeth only 9093, viz, 0.5 per cent., are filled, in other words, of 1000 carious teeth in school children, only five are filled. In an appendix comparative statistics are collected on the frequency of dental caries in adults and school children in German cities and rural districts, in Sweden, Norway, England, Scotland, Russia, Italy, Switzerland, Holland, Austria, Belgium, New Zealand, and Illinois. The bibliography contains no less than 206 references, the number of which could have been considerably increased, if the English and American publications had been given due consideration.

The present work is undoubtedly a most creditable one, and probably the most extensive that has so far been published on the subject of oral hygiene.—*Cosmos*, Feb. 1914.

*Administering Gas.* When using nitrous oxid, either alone or with oxygen, as a general anesthetic, have the patient clasp the hands together with the fingers interlacing. The contraction of the muscles during the stage of excitement will cause them to remain clasped, and the patient will be unable to hamper you by grabbing at the face-piece, your hands, or otherwise interfere with the operation.

If you are performing any lengthy operation, which necessitates your patient keeping the head in one position, raise or lower the head rest on the chair just an inch or so, every ten or fifteen minutes. The relief which the patient obtains from the strain on the neck muscles is greatly appreciated. —*New Jersey Dental Journal.*

*Gout and Pyorrhea Alveolaris.* Dr. Brubacher, of Munich, Germany, says in *Deutsche Monatsschrift*, that in the etiology of pyorrhea alveolaris, gout has been assigned an important role as a predisposing factor. Uric acid is supposed to be deposited in the peridental tissue, causing inflammation and preparing the ground for the invasion and growth of pyogenic bacteria. Arthritic diathesis has been emphasized as a cause of pyorrhea alveolaris, especially by American investigators. The fact, however, that a great many arthritics show no signs of pyorrhea has induced the writer to investigate the matter, by employing the murexid test upon superficial and deep deposits on extracted teeth of non-arthritics, arthritics, and patients suffering from arthritis deformans, as well as healthy persons. The murexid test, as every dental student knows, consists in adding to a small amount of the material to be examined a few drops of nitric acid on a porcelain dish, subjecting it to slow heat until dry, and allowing a drop of caustic ammonia to flow from the edge of the dish into the residue, whereupon a beautiful purple red is produced if uric acid is present. This test is an absolutely certain criterion for the presence of uric acid. If, however, albuminous substances are present in the substance to be tested, they may not only hide the purple red color of the murexid test by their yellow, orange-red, or red-brown coloration, viz, the xanthoproteic reaction, but may actually simulate that purple color. The controls made by the writer with the deposits collected from the teeth of healthy patients proved absolutely negative, but upon the addition of the slightest trace of uric acid, even in the presence of an abundance of proteids, the meruxid test produced the characteristic purple. After these preliminary tests, twenty-five tests were made with deposits gathered from arthritics, all of which showed negative. Neither did patients with pyorrhea alveolaris show any prevalence of arthritis upon clinical examination. The writer is consequently of the opinion that American investigators who believe that they have demonstrated uric acid in these cases have confused the proteid reaction with the murexid test, which mistake may easily occur if the porcelain dish be heated too rapidly and a trifle too much ammonia is added. According to the writer's investigations, the deposition of uric acid locally predisposes the surrounding tissues to the invasion of bacteria and protozoa no more than does any other dyscrasia which reduces vital cell energy.—*Dental Cosmos*, Feb., 1914, P. 241.

*Administration of Calcium.* To The Editor:—Please answer the following questions:

1. Which is the best salt of calcium to give intramuscularly or subcutaneously when you want calcium effect in case of calcium insufficiency? What about the irritation?

2. What are the dosage and dilution of solution of these salts when thus given?

3. Which is the best salt of calcium to give orally for an acidemia and for a calcium insufficiency?

4. What dosage orally?

5. Are the lactate and glycerophosphate equally good for a systemic effect of calcium for the nerves?

6. What foods have the largest percentage of calcium?

George F. Way, M.D., Buffalo, Ills.,

---

Answer.—1. The lactate of calcium is less irritating than the chlorid, and would probably be the best salt to give intramuscularly or by hypodermic injection. It is doubtful if the demand for calcium is ever so pressing as to require hypodermic injection.

2. A dose of 5 grains should be sufficient. Calcium, when administered internally, is rapidly excreted; hence there could be no object in giving large doses. It may be given in a 4 per cent. dilution.

3. The lactate and chlorid of calcium are commomly selected for internal administration. Of these the lactate is less irritating, and is preferable.

4. The dose is 0.5 gm., or 7½ grains.

5. Yes; the effect so far as the calcium is concerned will be the same. There is no good evidence that glycerophosphate has any special effect on the nerves or other functions.

6. Milk, yolk of egg, peas, egg-albumin, potato, wheat, beef. This order is determined by the proportion of calcium in the dried substance and would need some modification if we consider the foods in the form in which they are eaten.—*Journal, A. M. A.*

*The High Cost of Living As a Factor in Race Degeneracy.*

Dr. J. N. Hurty, Indianapolis: The high cost of living has a bearing on race degeneracy. If we could raise the price of diseased meats and whisky so high that they could not be bought, that would be an advantage. We place too much faith on the doctor and on medicine. We think that medicine can undo the wrong that we have done the body. It can only repair. It cannot remedy or remake. We must have medicine, but we must view it from a different angle. We are obsessed with the idea of cure. To eradicate from men's minds the idea that they ever can experience perfect cure after disease or injury would do a great deal of good. When we attempt to tell people that they must not get sick, and that getting sick is weakness, they go to the next doctor, who will give them a drop of medicine. Let us get away from that idea. —*Journal A. M. A.*

*A Hint Regarding The Use Of The Rubber Dam.*

The rubber is still necessary in many dental operations. Yet how many operators have ever stopped to think why the patients dislike it so much? If they would chew a piece of dam as it comes from the dental depot they would learn one reason, for rubber dam not only tastes badly, it also has a most unpleasant odor. Both these factors, evil taste and odor, as well as the general cleanliness of rubber dam, may be improved by simply washing it before use. For this purpose, first soap and water are employed; then the piece of dam is rinsed in clear water, and next washed in water to which a liberal amount of mouth-wash has been added. It is then hung up and allowed to dry. After the dam has thoroughly dried, a coat of borated talcum powder is dusted upon it, when the dam is ready for use. By chewing a small piece, the operator can readily convince himself of the marked improvement which this treatment imparts to the rubber.—*Ash's Monthly.*

*Soldering Backings to Platinum Pin Teeth.* Much trouble is sometimes caused through carelessness when backing teeth with gold or other metal. To avoid it, after the case has been waxed-up, invested and scalded out, it should be placed on an asbestos or fireclay slab and slightly heated up, until all the moisture has been driven off. The blowpipe flame should then be applied to the case and finally directed upon the solder.

If the case is allowed to remain upon the Bunsen beyond the drying-off stage, rapid and excessive oxidation takes place which is likely to be absorbed by the platinum pins and to render them brittle and very liable to fracture. —*Ash's Monthly.*

*The Importance of Frequent and Thorough Medical Examination of the Well.* Dr. Victor C. Vaughan, Ann Arbor, Mich.: Our country has a population of nearly one hundred millions. Millions of these are descent, respectable citizens —not altogether wise, but for the most part well-informed. Thousands are brutal in their instincts, criminal in their pursuits, and breeders of their kind. We assert that we are civilized, but there are those among us who would be stoned to death within less than twenty-four hours were they to attempt to live in a savage tribe. Decay and death come with advancing years. Those possessed of strength and health must bear the burden of those afflicted with poor health. The world has seen what has been done in Havana and the Canal Zone, where the most pestilential spots have been converted into healthful habitations. Scientific investigation has made these demonstrations, and the world applauds, but seems slow to make general application of the rules of hygiene. I look forward with confidence to the time when preventable diseases will be prevented, and when curable diseases will be detected and treated while curable.—*Journal A. M. A.*

*Treatment of Hydrofluoric Acid Burns.*

TO THE EDITOR:—What is the best treatment of burns made by hydrofluoric acid?

C. C. DUBOIS, M.D., Warsaw, Ind.

ANSWER:—The burns should be washed with diluted milk of magnesia. Ammonia is too caustic.—*Journal A. M. A.*

*Practical Eugenics.*

The extent to which the good and bad qualities of one generation are transmitted by heredity to another generation is an unsettled question; but there is no doubt that the conditions surrounding the fetus *in utero* have much to do with the vigor with which it enters on its extra-uterine life. Prenatal care of the mother is a logical extention of the philanthropy which is seeking to converse the vital resourses of the nation by the care of infants. Provision for such antenatal care has been made for the past four years by the Committe on Infant Social Service of the Women's Municipal League of Boston, and its example has been followed in a number of other cities. The committee now has made its report for the first four years of its existence with statistics for a part of the fifth year. The work of the committee has been restricted to the visiting of pregnant women once in ten days by a competent nurse, who takes blood-pressure, examines urine and advises regarding diet and regimen, but gives no drugs, with the exception of cascara. The succes of the work is judged by the decreasing frequency of eclampsia, stillbirths, premature births, and by the increase in the weight of the babies. In all these particulars they record a steady improvement as the result of prenatal care. Thus the figures for percentage of cases of threatened eclampsia for the first four years are, respectively, 10.2 per cent., 4.8 per cent., 1.7 per cent. and 0.0 per cent. The percentage of stillbirths fell from 2.6 per cent., the average for three years, to 1.7 per cent. for the fourth year. The corresponding figures for the premature births were 1.7 per cent. and 0.7 per cent. A slight increase in the average birth-weight is also recorded. These figures, the committee believes, present sufficient evidence of the effectiveness of prenatal care to justify the continuance of such work. The expense is slight, amounting to about three dollars a year per patient. It is intended to extend this treatment to private patients, the nurse reporting to the family physician instead of to the hospital, as is the case with the public work. —*Journal A. M. A.*

*Doctors Caught In An Antispitting Campaign.* The recent article by Stiles, concerning the carelessness of some physicians in matters of hygiene, has been attacked from various sources as being too caustic and as exaggerating the facts. In Chicago recently an antispitting campaign was inaugurated by the police and health departments. Instructions were given to the police to enforce one of the various antispitting ordinances. The results of a day's campaign are thus reported in one of the Chicago dailies:

Two physicians, Dr. O. H. B——— and Dr. A. H.———, were fined $2 and costs each by Judge Sabath yesterday for spitting. Seventy other men were fined $1 and costs. One was fined by Judge Dolan. Twenty-five were fined by Judge Goodnow in Hyde Park and three in Englewood. Dr. U. G. D——— spat on the floor of a State Street car at Forty-Seventh Street. A patrolman was riding on the car and arrested the physician.

On the other hand, it is reported that a conscientious policeman who caught himself in the act of expectoration took himself to the court and paid his fine.—*Journal A. M. A.*, Feb. 21.

*"Making Faces" As An Aid To Health.* Massage and exercise, at various times, have been advocated as a panacea for many and divers ills. Fernet now pro-proposes the idea that by exercise we may be able to influence favorably the encroaching deafness of the elderly, or that following otitis media. He remarks that children can often move the ears and part of the scalp voluntarily, and some may become quite exceptional in this faculty. Fernet believes that, through exercise, adults will be able to produce such movements. He has devised three series of exercises, as follows: The first consists of grimaces of the face, contracting in turn the muscles of the lips, nostrils and eyelids, aiming ultimately to reach the ear; next the frontal and occipital muscles are contracted alternately and then the muscles above and behind and in front of the ear. The muscles of the eustachian tube are then exercised by directions which he gives fully. He says, "Medical men, knowing the anatomy of the parts, can train their muscles very effectually in this way; others need an instructor to show them." Just what the ultimate outcome of this proposal will be is questionable. We may picture to ourselves tentatively a future period when all around us will be visible a series of grimacing, ear-wiggling elderly men and women in the act of improving their ears and warding off the encroaching deafness of old age. "Physicians, particularly," says Fernet, "need good hearing, and rather than accepting deafness with the fatalism of the Oriental, they should rise up and fight it." If it is to be combated by this method, the word "fight" is a very appropriate one.—*Journal A. M. A.*

*Condition of Blood in Hemophilia, etc.* Methods are described by Howell for testing the relative amount of prothrombin and antithrombin in the blood. The application of these methods to hemophilia shows that the blood in this condition is deficient in prothrombin. The antithrombin may be normal or somewhat greater than normal. The characteristic peculiarity of hemophilic blood in its markedly delayed time of coagulation. This peculiarity is explained by the diminution in amount of the prothrombin which results in a relatively excess of antithrombin. The detection of a hemophilic condition of the blood is facilitated by first oxalating the blood and then recalcifying with an optimum amount of calcium. Under these conditions the time of clotting exhibited by normal plasma is very constant (nine to twelve minutes). That of hemophilic blood is greatly delayed.

In patients suffering from spontaneous thrombosis of the veins Howell found evidence of a diminution in the antithrombin of the blood, the prothrombin being normal. It is suggested that this deficiency in antithrombin operates as a favoring, perhaps as a determining, factor in the production of the thrombus. In purpura hemorrhagica and other forms of so-called purpura, no evidence was found of any variation from normal in either the antithrombin or the prothrombin. —*Journal A. M. A.*

*Disinfection of the Hands.* To THE EDITOR:—1. What effect does prolonged scrubbing with soap and running water have as regards freeing the hands from the danger of carrying infection?

2. Are hands prepared thus and subsequently soaked for five minutes in alcohol safe for ordinary surgical procedures? RAY ERNEST SMITH, M.D., Rutland, Vt.

ANSWER.—Prolonged scrubbing removes the external layers of the epidermis and some of the bacteria There is a difference between practical results and technical laboratory results. Practical results show that proper washing with soap and water followed by scrubbing, not simply soaking, with from 70 to 80 per cent. alcohol gives clean hands, and that if this process be thoroughly followed no cultures will be made from surface contact, or in other words, from the surface of the hands; but if bits of skin be cut off from the hands so as to include the depth of the follicles, germs may be found which have been hidden in the depths of the follicles.

2. The reason that it is not desirable to operate with bare hands thus prepared is that the hands perspire and the use of the hands in handling instruments and tissues brings germs to the surface and thus makes it possible to infect the wound. While practical experience shows that the danger is very slight, yet the possibility of it makes it desirable not to operate without wearing properly sterilized gloves.

Notwithstanding the fact that experiments show that alcohol is not a germicide, practical results show that its proper use cleanses the hands better than any other method in use to-day. The trouble is that most surgeons have either had a little alcohol poured over the hands or dip the hands into the alcohol, both methods being useless. The hands must be scrubbed in order to be cleaned successfully. —*Journal A. M. A.*

*School Dental Dispensaries for Chicago.* The Chicago Health Department has provided in its 1914 Budget for the conduct of ten school dispensaries in various parts of the city. During the past year 26,655 school children were Dentally inspected, and only 2,321 were found normal. 26,929 treatments were given, 17,330 fillings were made and 13,348 teeth were extracted.

